# Effect of cooking on glycemic index, antioxidant activities, α‐amylase, and α‐glucosidase inhibitory properties of two rice varieties

**DOI:** 10.1002/fsn3.806

**Published:** 2018-10-11

**Authors:** Bukola C. Adedayo, Adeniyi A. Adebayo, Esther E. Nwanna, Ganiyu Oboh

**Affiliations:** ^1^ Functional Foods and Nutraceutical Research Unit Department of Biochemistry Federal University of Technology Akure Nigeria

**Keywords:** amylose/amylopectin ratio, glycemic index, rice varieties

## Abstract

This study was designed to investigate the influence of cooking on the glycemic index (GI), amylose, and amylopectin contents of two rice varieties. Two rice varieties (foreign long rice and ofada) were sourced for, divided into two, one portion cooked and the other used raw. The sugar, starch, amylose, and amylopectin contents as well as glycemic indices, antioxidant properties, and the ability of the rice to inhibit carbohydrate‐hydrolyzing enzymes (α‐amylase and α‐glucosidase) were determined. In addition, polyphenol content was determined. The results revealed that cooking caused a significant increase in starch content of the rice varieties. In the same vein, cooking increases the amylopectin content but has no effect on the amylose content. In addition, cooking shows no effect on polyphenol content but significantly increases radical scavenging ability of rice varieties used in this study. Furthermore, cooking lower the α‐amylase and α‐glucosidase inhibitory properties of two rice varieties except for foreign rice. However, the inhibitory effect of both cooked and raw foreign and ofada rice on α‐amylase and α‐glucosidase coupled with their low GI may explain their important role in controlling blood glucose level.

## 1 INTRODUCTION

Generally, carbohydrates are sole source of energy in human nutrition. Some years back, hyperglycemia and obesity play front role in health problems affecting many lives. Carbohydrates are not only sources of energy, but certain types of carbohydrate are included in the diet depending on the prevailing physiological condition (Deepa, Singh, & Naidu, 2008). Intake of whole grain diet has gained popularity in recent years due to its health beneficial effects especially in the prevention of chronic diseases such as cardiovascular diseases, type 2 diabetes, and cancer among others because of present awareness of alterations in the glycemic index (GI) followed the consumption of carbohydrate‐rich food (Fardet, 2010; Marquart, Slavin, & Fulcher, 2002). Whole grains are important source of dietary fiber that encloses numbers of bioactive compounds and nutrients (Deepa et al., 2008; Marquart et al., 2002). Unhydrolyzed starch also known as resistant starch is the residual portions of starch—resistant to enzyme hydrolysis—entering the large intestine in conjunction with dietary fiber. Unhydrolyzed starch only accounts for little portion of total calorie intake; however, its role is similar to those of other fiber components (Qi et al., 2006).

Rice (*Oryza sativa*) is the most consumed staple food in nearly all human population (Hu, Zhao, Duan, Linlin, & Wu, 2004). In Nigeria, rice represents main staple food among people because of its low cost and quick way of preparation. In recent years, there has been considerable keen interest on the strong correlation between type 2 diabetes and rice consumption (Hu, Pan, Malik, & Sun, 2012). The concept of GI was first explained by Jenkins in 1981, who ranked different foods based on their glycemic response (Jenkins et al., 1981). GI was described as indices for monitoring postprandial blood glucose with respect to intake of carbohydrate‐rich foods (Jenkins et al., 1981). GI is based on carbohydrate absorption rate principle. The lower the GI, slower the rate of carbohydrate absorption and the lower the postprandial glucose level (Brand et al., 1991). Persistent rise in blood glucose level can cause cellular damage, and this has been interrelated with pathogenesis of many diseases (Kwon, Apostolidis, & Shetty, 2008; Sunyer, 2002). Hence, there is scientific interest in the various roles play by low GI foods in controlling chronic diseases.

Previous study reported the association between the radical scavenging ability of foods rich in antioxidant and their efficacy in the management of degenerative diseases such as diabetes and cardiovascular diseases (Oboh et al., 2014). It has been reported that cooking caused a significant alteration in chemical compositions as well as antioxidant activities of grains and cereals (Pellegrini et al., 2010). Alpha‐amylase and alpha‐glucosidase play crucial role in sudden rise in blood glucose level observed in diabetic patients after taking carbohydrate‐rich foods due to hydrolysis of carbohydrate by these enzymes into simpler monomeric unit (glucose) (Kim, Jeong, Wang, Lee, & Rhee, 2005; Kwon, Vattem, & Shetty, 2006). Hence, attenuation of these enzymes involved in the hydrolysis of starch can lower the postprandial increase in blood glucose after eating carbohydrate‐rich food and this could be of importance in the management of type 2 diabetes. Generally, grains undergo various processing methods (cooking, frying, roasting, etc.) before consumption and these processing techniques have effect on the concentration and bioavailability of some vital nutrients and compounds. Previous studies showed that cooking significantly reduced total phenolic contents of some grains (Pellegrini et al., 2010; Towo, Svanberg, & Ndossi, 2003). Having in mind the lack of scientific literatures on the effect of cooking on in vitro GI, α‐amylase, and α‐glucosidase inhibitory properties of grains, therefore, present study was designed to assess the effect of cooking on amylose and amylopectin contents, sugar and starch contents, GI, and antioxidant properties of two rice varieties, as well as their effects on starch hydrolyzing enzymes (α‐amylase and α‐glucosidase).

## 2 MATERIALS AND METHODS

### 2.1 Sample collection and preparation

Two different rice varieties (foreign long rice and ofada rice) were collected from Akure main market, Akure, Nigeria. For cooked samples, 400 g of each rice sample was weighed and cooked for about 30 min. The cooked samples were drained and freeze‐dried. The dried samples (raw and cooked) were milled to powder and kept dry before extraction. Five grams of the powdered rice sample was weighed and soaked in 100 ml of distilled water for 24 h and thereafter filtered through Whatman no 1 filter paper; the filtrate was kept in refrigerator and later used for the assays.

### 2.2 Chemicals and reagents

Chemicals such as dinitrosalicylic acid, p‐nitrophenyl‐α‐D‐glucopyranoside, hog pancreatic α‐amylase, and α‐glucosidase were obtained from Sigma‐Aldrich, Inc. (St. Louis, MO, USA). Acetic acid, gallic acid, quercetin, sulfuric acid, sodium carbonate, potassium acetate, phenol, sodium hydroxide, and perchloric acid were procured from BDH Chemicals Ltd. (Poole, Dorset, UK). All other chemicals used in this study were of analytical grade, and glass‐distilled water was used.

### 2.3 Quantitative phytochemical analysis

The total phenolic content of raw and cooked rice was determined using the Folin–Ciocalteu method as described by Singleton, Orthofor, and Lamuela‐Raventos (1999). In total phenolic content determination, gallic acid was used as standard and result was expressed as milligram gallic acid equivalent per gram of dry sample (mgGAE/g). Furthermore, total flavonoid content was carried out according to the standard method of Meda, Lamien, Romito, Millogo, and Nacoulma (2005), using quercetin as standard.

### 2.4 Determination of antioxidant activities

The antioxidant activities of both raw and cooked rice varieties were assessed through radical scavenging ability of the extracts. 1, 1‐diphenyl‐2‐picrylhydrazyl (DPPH) radical scavenging ability of rice extracts was determined using the previously described method of Gyamfi, Yonamine, and Aniya (1999). In addition, 2, 2′‐azino‐bis 3‐ethylbenzthiazoline‐6‐sulfonic acid (ABTS) radical scavenging ability of the extracts was determined according to the previously described method of Re et al. (1999). Furthermore, ferric reducing antioxidant property of the extracts was assessed according to the standard method of Oyaizu (1986).

### 2.5 α‐amylase inhibition assay

In assessing the α‐amylase inhibitory effect of the extracts, appropriate dilution of cooked and raw rice extracts and Hog pancreatic amylase (0.5 mg/ml) in 0.02 M sodium phosphate buffer (pH 6.9, containing 0.006 M NaCl) were incubated at 25°C for 10 min. Then, 1% starch solution in 0.02 M sodium phosphate buffer (pH 6.9) was added. The reaction mixture was incubated at 25°C for 10 min and terminated with addition 1.0 ml of dinitrosalicylic acid. Thereafter, the mixture was incubated in a boiling water bath for 5 min and cooled at room temperature. The reaction mixture was then diluted by addition of 1 ml distilled water, and absorbance was measured at 540 nm using spectrophotometer. The α‐amylase inhibitory effect of the extracts was subsequently calculated and expressed as percentage inhibition (Worthington, 1993).

### 2.6 α‐glucosidase inhibition assay

In brief, appropriate dilutions of the extracts and α‐glucosidase solution (1.0 U/ml) in 0.1 M phosphate buffer (pH 6.9) were incubated 25°C for 10 min, and then, 100 μl of 5 mM p‐nitrophenyl‐α‐D‐glucopyranoside (PNPG) solution in 0.1 M phosphate buffer(pH 6.9) was added. The mixture was incubated at 25°C for 5 min, and the absorbance was read at 405 nm using spectrophotometer. The α‐glucosidase inhibitory effect of the extracts was calculated and expressed as percentage inhibition (Apostolidis, Kwon, & Shetty, 2007).

### 2.7 In vitro glycemic index

In vitro starch hydrolysis rate and GI were determined according to Goni, Garcia‐Alonso, and Saura‐Calixto (1997). Fifty milligrams of freeze‐dried sample was incubated with 1 mg of pepsin in 10 ml HCl‐KCl buffer (pH 1.5) at 40°C for 60 min in a shaking water bath. Digested samples were diluted with 2.5 ml phosphate buffer (pH 6.9), and then, 5 ml of α‐amylase solution (in phosphate buffer) was added. The mixture was incubated at 37°C in a shaking water bath, and 0.1 ml was taken from each flask every 30 min from 0 to 3 h and boiled for 15 min to inactivate the enzyme. Sodium acetate buffer (0.4 M, pH 4.75) was added, and the residual starch was digested to glucose by adding 3 ml α‐glucosidase and incubating at 60°C for 45 min. Glucose concentration was quantified by adding 200 ml of dinitrosalicylic acid color reagent. The reaction mixture was stopped by boiling the mixture in a water bath for 5 min and then cooled to room temperature. The mixture was further diluted by addition of 5 ml distilled water and centrifuged at 1,200 *g*. The supernatant was collected, and the absorbance was read at 540 nm using spectrophotometer. The rate of starch digestion was expressed as the percentage of starch hydrolyzed per time.

### 2.8 Determination of soluble sugar and starch

Soluble sugar and starch were determined according to the methods of Williams, Wu, Tasi, and Bates (1958) and Juliano (1971). For free sugar analysis, 50 mg of freeze‐dried sample was weighed into the beaker, 0.5 ml of 80% ethanol was added to the sample and stirred, 10 ml distilled water was added to the mixture and shaken, 5 ml of hot ethanol was added and mixed thoroughly, the mixture was centrifuged and supernatant was decanted into a 50‐ml flask, another 5 ml hot ethanol was added to the residue and centrifuged for another 5 min, and the supernatant was added into the same flask. The residue was kept for starch analysis. Standard was added to the extract, distilled water, 5% phenol was added to the extract, and concentrated H_2_SO_4_ was also added; the absorbance of the mixture was taken at 450 nm using spectrophotometer. For starch analysis, the residue from the sugar analysis was rinsed with 9 ml of 70% per chloric acid into 50‐ml standard flask; the tube was rinsed into the flask with 4.5 ml distilled water. It was allowed to stand for 90 min, the mixture was made up to 50 ml with distilled water, the mixture was filtered through glass wool, 0.2 ml of the filtrate was pipetted into test tube, and 1.8 ml of distilled water was added. The standard solution was added to the extract; distilled water, 5% phenol, and concentrated H_2_SO_4_ were added; and the absorbance reading was taken at 450 nm.

### 2.9 Determination of amylose and amylopectin content

Hundred milligrams (100 mg) of each samples was weighed into 100‐ml volumetric flask. Then, 1 ml of 95% ethanol and 9 ml of 1 N NaOH were carefully added and samples were heated for 10 min in a boiling water bath to gelatinize the starch. The mixture was cooled and make up to volume with water. A 5 ml portion of the starch solution was measured into a 100‐ml standard flask, and then, 1 ml of 1 N acetic acid and 2 ml of iodine solution were added. This was then made up to volume with distilled water. Thereafter, the mixture was shaken and absorbance was measured at 620 nm after 20 min. Amylopectin content was derived from starch and amylose content gotten to difference (Juliano, 1971; Williams et al., 1958).

### 2.10 Determination of EC_50_ values

Where applicable, EC_50_ (extract concentration causing 50% inhibition in the activities of enzyme) value was calculated using nonlinear regression analysis.

### 2.11 Data analysis

The results of triplicate experiments were analyzed and expressed as mean ± standard deviation (SD). Mean was compared by one‐way analysis of variance (ANOVA) followed by Duncan's multiple range test, and least significant differences were carried out accepted at *p *<* *0.05.

## 3 RESULTS

The result of soluble starch contents of raw and cooked foreign and ofada rice is presented in Table 1. The results revealed that cooking caused a significant increase in the starch content of two rice varieties, foreign rice (27.9 g/100 g) and ofada rice (25.5 g/100 g) when compared with raw samples, foreign rice (23.4 g/100 g) and ofada (23.4 g/100 g). Similarly, the result of the effect of cooking on the GI of two rice varieties (foreign and ofada) is presented in Table 1. The results showed that cooking slightly altered the GI of two rice varieties used in this study: raw [foreign rice (48.4%) and ofada rice (49.2%)] and cooked [foreign rice (49.0%) and ofada rice (51.8%)]. The effect of cooking on the amylose and amylopectin contents is presented in Table 2. As shown in Table 2, cooking increases the amylopectin content but has no effect on the amylose content of two rice varieties.

**Table 1:** Effect of cooking on amylose, amylopectin, and amylose/amylopectin ratio of two rice varieties

Sample	Amylose (g/100 g)	Amylopectin (g/100 g)	Amylose/amylopectin ratio
Foreign rice			
Raw	3.07 ± 0.56^a^	24.59 ± 2.51^c^	0.125
Cooked	3.13 ± 0.61^a^	20.33 ± 1.92^a^	0.154
Ofada rice			
Raw	3.07 ± 0.71^a^	22.33 ± 1.08^b^	0.137
Cooked	3.19 ± 0.68^a^	20.33 ± 1.71^a^	0.157

Values represent mean ± standard deviation of replicate experiments. Values with the same superscript alphabet along the same column are not statistically different (*p *<* *0.05).

**Table 2:** Effect of cooking on starch, sugar, and glycemic index of two rice varieties

Sample	Starch (g/100 g)	Sugar (g/100 g)	Glycemic index (%)
Foreign rice			
Raw	23.40 ± 2.31^a^	2.77 ± 0.70^a^	48.40 ± 3.67^a^
Cooked	27.90 ± 1.93^c^	3.13 ± 0.20^b^	49.00 ± 4.03^a^
Ofada rice			
Raw	23.10 ± 3.11^a^	2.77 ± 0.71^a^	49.20 ± 2.99^a^
Cooked	25.50 ± 0.97^b^	3.31 ± 0.99^b^	51.80 ± 3.01^b^

Values represent mean ± standard deviation of replicate experiments. Values with the same superscript alphabet along the same column are not statistically different (*p *<* *0.05).

Table 3 shows the effect of cooking on the phenolic contents, ferric reducing antioxidant property, and ABTS radical scavenging ability of two rice varieties. The results revealed that cooking has no effect on total phenol, total flavonoid, and ferric reducing antioxidant property, whereas it significantly increases the ABTS radical scavenging ability of two rice varieties.

**Table 3:** Effect of cooking on total phenol, total flavonoid, ferric reducing antioxidant property (FRAP), and ABTS radical scavenging ability of two rice varieties

Samples	Total phenol	Total flavonoid	FRAP	ABTS*
Foreign rice				
Raw	34.1 ± 1.0^a^	14.0 ± 1.1^a^	13.3 ± 0.0^a^	26.3 ± 1.2^b^
Cooked	38.0 ± 3.1^a^	15.2 ± 0.0^a^	17.2 ± 1.4^b^	31.1 ± 2.1^c^
Ofada rice				
Raw	35.2 ± 1.1^a^	15.1 ± 2.1^a^	15.5 ± 2.3^b^	19.3 ± 3.1^a^
Cooked	37.3 ± 2.0^a^	16.4 ± 3.2^a^	16.0 ± 1.5^b^	25.2 ± 2.7^b^

Values represent mean ± standard deviation of replicate experiments. Values with the same superscript alphabet along the same column are not statistically different (*p *<* *0.05). Total phenol in mgGAE/100 g, total flavonoid in mgQUE/100 g, FRAP in mgAAE/100 g, and ABTS radical scavenging ability in mmolTEAC/g.

**Figure 1:**
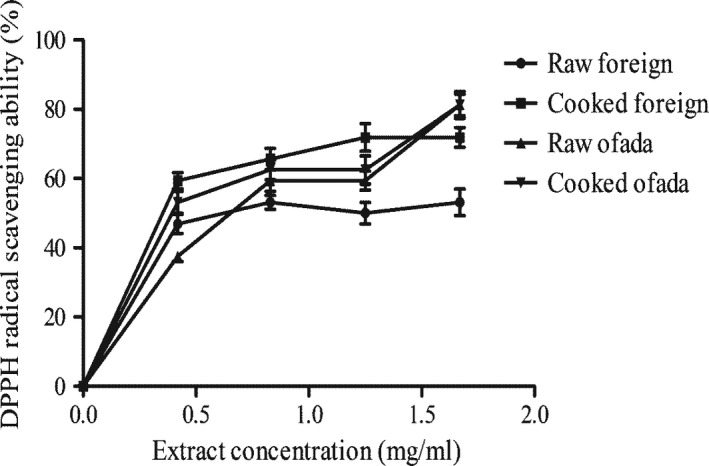
Effect of cooking on DPPH radical scavenging ability of two rice varieties

Figure 1 shows the effect of cooking on DPPH radical scavenging ability of two rice varieties. The results revealed that samples scavenged DPPH radical in a concentration‐dependent manner. However, cooking significantly increases DPPH radical scavenging ability of the two rice varieties (Table 4).

**Table 4:** IC_50_ values of effect of cooking on DPPH radical scavenging ability, α‐amylase, and α‐glucosidase inhibitory properties of two rice varieties

Samples	α‐amylase (mg/ml)	α‐glucosidase (mg/ml)	DPPH radical (mg/ml)
Foreign rice			
Raw	0.99 ± 0.05^a^	2.59 ± 0.61^b^	1.19 ± 0.38^c^
Cooked	1.37 ± 0.06^b^	1.91 ± 0.65^a^	0.73 ± 0.03^a^
Ofada rice			
Raw	1.43 ± 0.06^b^	2.64 ± 0.90^b^	0.89 ± 0.02^b^
Cooked	1.89 ± 0.07^c^	3.85 ± 0.10^c^	0.78 ± 0.04^a^

Values represent mean ± standard deviation of replicate experiments. Values with the same superscript alphabet along the same column are not statistically different (*p *<* *0.05).

**Figure 2:**
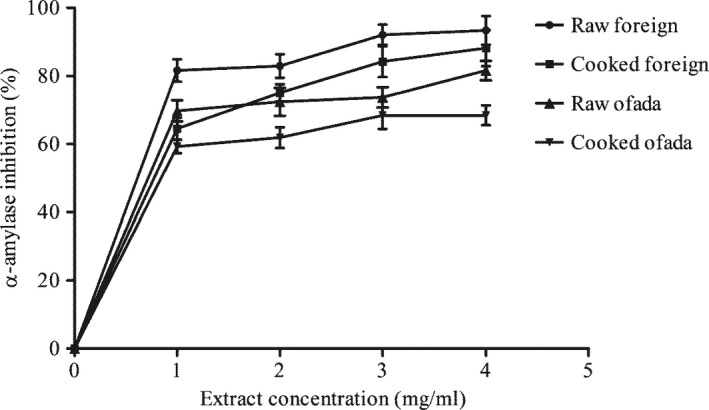
Effect of cooking on α‐amylase inhibitory property of two rice varieties

**Figure 3:**
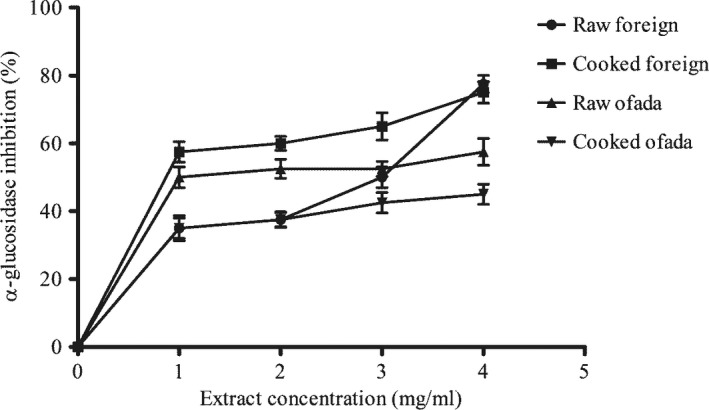
Effect of cooking on α‐glucosidase inhibitory property of two rice varieties

Furthermore, α‐amylase inhibitory effect and α‐glucosidase inhibitory effect of cooked and raw rice varieties are presented in Figures 2 and 3, respectively. As shown in Table 4, cooking reduced the α‐amylase and inhibitory property of two rice varieties. Conversely, cooking increased the α‐glucosidase inhibitory activity of foreign rice but lowered its α‐amylase inhibitory property.

## 4 DISCUSSION

Plant foods are consumed by man and often times; they are generally processed before consumption. Some of the processing methods include cooking, boiling, frying, roasting, drying, and baking among others. However, these treatments are capable of influencing the glycemic indices of the food (Ludwig, 2002). Recently, studies have emphasized the role played by processing method as they are capable of modifying the in vivo starch bioavailability (Borget, 1992). Structure and digestibility of starchy food are affected by processing methods, and this could influence glycemic response (Lehmann & Robin, 2007).

In this study, effect of cooking on the GI of two rice varieties was evaluated. As revealed in this study, cooking caused a slight increase in the GI when compared with the raw samples. The increased GI of cooked rice may be explained by the association between high temperature and humidity, which modify the physico‐chemical properties of the varieties. Interestingly, starch granules take up water and swell during cooking (i.e., gelatinization), which irreversibly disrupts the crystalline structure of the starch, making it susceptible to hydrolysis by α‐amylase (Englyst, Quigley, & Hudson, 1995; Soh & Brand‐Miller, 1999). Nevertheless, the variation in GI observed in the two varieties may be due to the quality of starch present in each rice varieties. GI depends on the carbohydrates consumed in foods on the basis of blood glucose level after consumption of such foods (Jenkins et al., 1981, 2002). Changes in diet are important in the control of type 2 diabetes, with/without insulin involvement. The concept of GI was proposed with the aim of assisting diabetic subjects in selecting their foods, with recommendation that foods with low GI are good for diabetic patients (Thorne, Thompson, & Jenkins, 1983). Using glucose as standard, foods can be classified into low (≤55), medium (55–69), and high (≥70) GI (Jenkins et al., 1981). The low GI of rice varieties used in this study could be attributed to their polyphenol and fiber. Fibers convert intestinal contents into gel‐like substance that retard enzymatic activity on starch, thereby resulted into low GI (Oh et al., 2005). Howlett and Ashwell (2008) reported that low GI foods improve glycemic control by lowering glycated end‐products and improving insulin sensitivity. However, GI does not measure the quantity of carbohydrate, but its quality (Foster‐Powell, Holt, & Brand‐Miller, 2002). Although cooking increased GI of ofada rice, the GI value reported in this study was comparable to that of Barakatun‐Nisak, Ruzita, and Norimah (2005).

Furthermore, the apparent increase in the starch content of the rice grains after cooking could be due to retrogradation. Gelatinized starch is not thermodynamically stable, hence a progressive re‐association of the starch molecules upon cooling (Torres, Tena, Murray, & Sarkar, 2017). This recrystallization is referred to as retrogradation and may be responsible for the apparent increase in the starch content after cooking (Garcia‐Alonso, Jimenez‐Escrig, Martin‐Carron, Bravo, & Saura‐Calixto, 1999). In addition, cooking also leads to swelling up of starch granules, which exposes the starch chain and making them susceptible to activities of hydrolyzed enzymes (α‐amylase and α‐glucosidase).

High level of amylose in food products has been related to low blood glucose in comparison with high amylopectin food products. Findings in this study revealed that amylopectin level is higher than amylose content still the rice samples displayed a moderate GI values. This discrepancy could be attributed to the presence of other components such as fiber and polyphenols, which have been previously described to lower blood glucose (Salmeron, Manson, Stampfer, Colditz, & Wing, 1997). This agrees with previous report that rice varieties with similar amylose content could have different digestibility possibly due to their physico‐chemical properties (Panlasigui et al., 1991). The amylose content of cooked and raw rice varieties reported in this study was lower than the amylose content of white rice cultivated in Thailand (Sompong, Siebenhandl‐Ehn, Berghofer, & Schoenlechner, 2011). The differences could be due to effect of geographical location. However, regardless of their amylose content, the two rice varieties used in this study both cooked and uncooked have moderate GI values.

In one of studies carried out by Jenkins and his colleagues, they proposed that the concept of GI is no longer genuine as far as management of diabetes is concern. They further proposed that pharmacological approach to lower the carbohydrate absorption, possibly through inhibition of enzymes (α‐amylase and α‐glucosidase) that hydrolyze carbohydrate is of importance in the management of diabetes especially type 2 diabetes (Jenkins et al., 2002). Interestingly, findings in this study revealed that both foreign and ofada rice used in this study inhibited α‐amylase and α‐glucosidase. α‐Amylase and α‐glucosidase are important enzymes in carbohydrate metabolism, and inhibition of these enzymes could be important in reducing the amount of glucose released into the system and thereby influencing GI. This forms the pedestal of the proposed mechanism of action of inhibitors of α‐amylase and α‐glucosidase in reducing GI. Pancreatic α‐amylase and intestinal α‐glucosidase play important role in starch hydrolysis and glucose uptake (McDougall & Stewart, 2005). Evaluation of effect of rice extracts on the activity of α‐amylase and α‐glucosidase showed a concentration‐dependent relationship. Cooking increased α‐amylase inhibitory potential of rice varieties used in this study. This agreed with earlier study by Donkor, Stojanovska, Ginn, Ashton, and Vasiljevic (2012) where some cereals inhibited α‐amylase and α‐glucosidase activity in vitro.

The rice varieties used in this study demonstrated strong radical scavenging ability as revealed by their effect on radicals such as ABTS and DPPH in vitro. The radical scavenging activities of the rice samples could be attributed to their polyphenol contents. This finding agreed with earlier studies where ability of plant foods to scavenge‐free radical correlates with their phenolic contents (Oboh et al., 2015). The most practical application of plant‐based foods is in the management of diseases. Therefore, regular supply of dietary antioxidants to enhance body defense mechanisms could be one of practical approaches to the prevention and/or management of chronic diseases. In addition, cooking enhanced radical scavenging activities of rice varieties used in this study. In agreement with our findings, Sharma and Gujral (2010) processed barley cultivars showed higher DPPH radical scavenging than raw barley.

## 5 CONCLUSION

The results of this study showed that cooking increased the antioxidant activities, glycemic indices, and enzyme inhibitory properties of two rice varieties (ofada and foreign), but has no effect on their phenolic contents. However, the rice varieties used in this study could be categorized as low GI food. These results have provided necessary information for the effective utilization of rice as functional food materials for controlling blood glucose level.

## ETHICAL STATEMENT

All guidelines for the care and use of animals were strictly adhered.

## CONFLICT OF INTEREST

Authors have no conflict of interest to declare.
